# 

**Published:** 1876-07

**Authors:** 


*The Dental Society of the State of New York* will hold its eighth annual meeting in the capitol at Albany, on Wednesday, Thursday and Friday, July 12th, 13th and 14th, 1876.

It is hoped that members will come fully prepared to discuss the subjects selected by the essayists, and to report "incidents of office practice," or the results of special lines of research and experiment, to which a portion of each day will be appropriated.

Members desiring to present voluntary papers will please notify O. E. Hill, 160 Clinton Street, Brooklyn, at as early a date as possible.

The Board of Censors meet on Tuesday, July 11th, in the Capitol, for the examination of all candidates who present themselves for the diploma of the Society conferring the degree of "Master of Dental Surgery" (M. D. S.) Those desiring further information concerning this examination should address F. French, Rochester, the Secretary of the Board.

Delegates from several State Societies are expected to be present, and visitors from kindred organizations in this or other States will be cordially received.

Members and others in attendance at this meeting are advised that accommodations are generously offered them by the proprietors of the Delavan House at reduced rates.

---

### *American Academy of Dental Science.*

The ninth annual meeting of the American Academy of Dental Science will be held in Boston, on Monday, September 25th, 1876, at 10 o'clock A. M.

The annual address will be delivered at 2 P. M., by Dr. Robert Arthur, of Baltimore.

E. N. Harris,
*Corresponding Secretary.*

---

### SELECTED ARTICLES.

### TO READERS AND CORRESPONDENTS.

Communications intended for publication, and exchange journals and papers, should be addressed to the Editor of the American Journal of Dental Science, 259 N. Eutaw St., Baltimore, Md. All letters relating to the business of the journal, or containing cash remittances, to be directed to Messrs. Snowden & Cowman. Articles for publication should be received by the editor at least three weeks previous to the first day of the month on which the number in which it is designed for them to appear, is issued.

### CONTENTS:

### ORIGINAL COMMUNICATIONS.

### MONTHLY SUMMARY.

☞The American Journal of Dental Science will contain fifty pages of reading matter in each number, making a volume of not less than

*Six Hundred* pages

**EXCLUSIVE OF ADVERTISEMENTS**

THE

### AMERICAN JOURNAL

OF

### DENTAL SCIENCE.

F. J. S. GORGAS, M. D., D. D. S., Editor.

The *Journal* is issued on the first of each month and contains not less than *fifty* pages of reading matter in each number, exclusive of advertisements, making a volume of *six hundred* pages per annum.

In each number will be found Original Communications, Correspondence, Selected Articles, Monthly Summary, Bibliographical Notices and Editorial Matter, and every effort will be exerted to render the *Journal* worthy of the patronage of the Dental profession.

A generous co-operation is respectfully solicited from the members of the profession; and as the *Journal* has now a printing office of its own, which materially lessens the cost of its publication, it will be issued at the reduced subscription price of

**$2.50 Per Annum in Advance.**

Communications are respectfully solicited on all subjects pertaining to the practice of dentistry, as well as reports of cases occurring in dental practice.

### RATES OF ADVERTISING.

| | 1 MO. | 2 MO. | 3 MO. | 6 MO. | 12 MO. |
|---|---|---|---|---|---|
| 1 PAGE. | $15 00 | $22 50 | $30 00 | $50 00 | $100 00 |
| ½ " | 10 00 | 15 00 | 20 00 | 30 00 | 50 00 |
| ¼ " | 7 00 | 11 00 | 15 00 | 20 00 | 30 00 |
| ⅛ " | 5 00 | 8 00 | 11 00 | 15 00 | 20 00 |

All original communications designed for publication, works for review, new instruments for notice, exchanges, &c., should be directed to the editor, 259 N. Eutaw St., Baltimore, Md.

All advertisements and subscriptions should be addressed to

SNOWDEN & COWMAN,

*Publishers of the Am. Journal of Dental Science.*

86 W. FAYETTE ST. BET. CHARLES & LIBERTY STS.,

BALTIMORE, MD.

